# Harmful Effects of the Azathioprine Metabolite 6-Mercaptopurine in Vascular Cells: Induction of Mineralization

**DOI:** 10.1371/journal.pone.0101709

**Published:** 2014-07-16

**Authors:** Jasmin Prüfer, Mirjam Schuchardt, Markus Tölle, Nicole Prüfer, Matthias Höhne, Walter Zidek, Markus van der Giet

**Affiliations:** 1 Charité – Universitaetsmedizin Berlin; Charité Centrum 13, Department of Nephrology Campus Benjamin Franklin, Berlin, Germany; 2 Freie Universitaet Berlin, Fachbereich Biochemie, Chemie, Berlin, Germany; 3 Universitaet Potsdam, Department of Nutrition Science, Potsdam, Germany; 4 Novartis Germany GmbH, Nuernberg, Germany; University of California, Los Angeles, United States of America

## Abstract

Vascular mineralization contributes to the high cardiovascular morbidity and mortality in patients who suffer from chronic kidney disease and in individuals who have undergone solid organ transplantation. The immunosuppressive regimen used to treat these patients appears to have an impact on vascular alterations. The effect of 6-mercaptopurine (6-MP) on vascular calcification has not yet been determined. This study investigates the effect of 6-MP on vascular mineralization by the induction of trans-differentiation of rat vascular smooth muscle cells *in vitro*. 6-MP not only induces the expression of osteo-chondrocyte-like transcription factors and proteins but also activates alkaline phosphatase enzyme activity and produces calcium deposition in *in vitro* and *ex vivo* models. These processes are dependent on 6-MP-induced production of reactive oxygen species, intracellular activation of mitogen-activated kinases and phosphorylation of the transcription factor Cbfa1. Furthermore, the metabolic products of 6-MP, 6-thioguanine nucleotides and 6-methyl-thio-inosine monophosphate have major impacts on cellular calcification. These data provide evidence for a possible harmful effect of the immunosuppressive drug 6-MP in vascular diseases, such as arteriosclerosis.

## Introduction

Pathological vascular changes and the subsequent high incidence rates of non-fatal and fatal cardiovascular events are major complications in patient cohorts with chronic renal failure and in patients who have undergone successful kidney transplantation [1], [2]. The intima and media of the vessel wall may be involved in mineralization, and changes at both sites can coexist. Cardiovascular morbidity and mortality can increase due to hemodynamic differences in the stiffened vessel [2]. Risk factors that promote arteriosclerosis are similar to those known to cause atherosclerosis and include high arterial blood pressure, diabetes mellitus, advanced age and chronic kidney disease (CKD) [3]–[6]. Furthermore, there are several indications that oxidative stress enhances the progression of vascular mineralization and arteriosclerosis [7], [8].

At present, arteriosclerosis is validated as an active cell-regulated process. One hypothesis for this process postulates that a phenotypic transformation of vascular smooth muscle cells (VSMCs) occurs. VSMCs are transformed into an osteo-chondrogenic cell (OCC) phenotype. The cells then express OCC-specific proteins, such as core-binding factor alpha 1 (Cbfa1) or alkaline phosphatase (ALP) [9]. The OCCs then begin to release calcium and phosphate, forming extracellular hydroxyapatite crystals. This process is commonly referred to as calcification [6].

After successful renal transplantation, cardiovascular mortality is lower compared to patients with end-stage renal disease, but mortality is still increased compared to an age-matched population with normal kidney function [1], [10]. recent longitudinal study elucidated that vascular calcification in kidney transplant patients is substantial within four years [11]. There is a growing body of direct and circumstantial evidence that immunosuppressive therapy might influence the progression of vascular alterations by affecting signaling pathways in multiple cell types [1]. Some reports describe the influence of immunosuppressive drugs on the overall cardiovascular outcome [1], [12], [13], but only limited knowledge is available concerning the influence of immunosuppressive drugs on arteriosclerosis progression.

Among the commonly used immunosuppressive drugs investigated, 6-mercaptopurine (6-MP) has the most prominent effect on *in vitro* calcification. Here, we report that 6-MP induces mineralization of VSMCs in *in vitro* and *ex vivo* models. 6-MP stimulation leads increased calcium deposition resulting from its capacity to induce trans-differentiation of VSMCs into cells expressing typical OCC markers. The induction of oxidative stress by 6-MP and its metabolites is a major contributor to the calcifying phenotype of VSMCs. The data reveal a possible harmful effect of 6-MP treatment that may enhance the progression of arteriosclerosis.

## Methods

Please see Methods S1 for a detailed description of the methods and protocols.

### Animals and cell culture

All animal experiments were conducted under the guidelines of the Protection of Animals. The protocol was approved by the Berlin Ethics Committee of Animal Experiments, The Landesamt für Gesundheit und Soziales, Berlin, Germany (permit number O0212/02). Preparation of the aorta from Wistar rats was accomplished under sodium pentobarbital anesthesia (400 mg/kg body weight, intraperitoneal injection). All efforts were made to minimize animal suffering. The adventitia of the rat aorta was removed before further procedures were performed. The outgrowth technique was used for VSMC isolation from the rat aorta [14]. The aorta was cut into rings for *ex vivo* assays.

Human VSMCs were purchased from PromoCell, where organ preparation conformed to the Declaration of Helsinki. Cells in passages 3 to 8 were used for experiments.

### Induction of *in vitro* and *ex vivo* calcification

*In vitro* calcification of rat VSMCs and *ex vivo* calcification of rat aortic rings were induced as described previously [15], [16]. The initial day of culture in CM (10 mmol/L β-glycerophosphate, 284 µmol/L ascorbic acid and 10 mmol/L sodium pyruvate) was defined as day 0. The medium was replaced every 3 days.

### Cell stimulation

Aortic rings and VSMCs were stimulated for short-term (24–48 hours) or long-term (up to 21 days) experiments. In short-term experiments using antagonists, cells were pre-treated for 30 min with each antagonist.

### Detection of mineralization

Alizarin Red staining was used to detect the mineralization status of the aortic rings and VSMCs, and quantification of the calcium content was determined either using the o-cresolphthalein method or by measuring the ALP activity.

### mRNA expression

After stimulation, cells were harvested for RNA isolation, reverse transcription was performed, and mRNA expression was determined via quantitative real-time PCR. mRNA expression values were normalized to those of the house keeping gene β-actin.

### Western blot

Protein lysates of VMSCs were separated into cytosolic and nuclear fractions. The nuclear protein fraction was separated by electrophoresis on a polyacrylamide gel, transferred to a polyvinylidene difluoride membrane, and incubated with specific antibodies, including anti-Cbfa1, anti-Cbfa1-phospho-Ser^465^ and anti-TATA-binding protein. For protein quantification, band intensities of 3 blots from independent experiments were analyzed using Bio1D-software (Vilber Lourmat).

### Statistical analysis

Results are presented as means±SEM, and statistical significance was determined by a Mann-Whitney test, unless otherwise indicated. A P value <0.05 was considered statistically significant.

## Results

### 6-MP enhances mineralization *in vitro* and *ex vivo*

**Figure 1:**
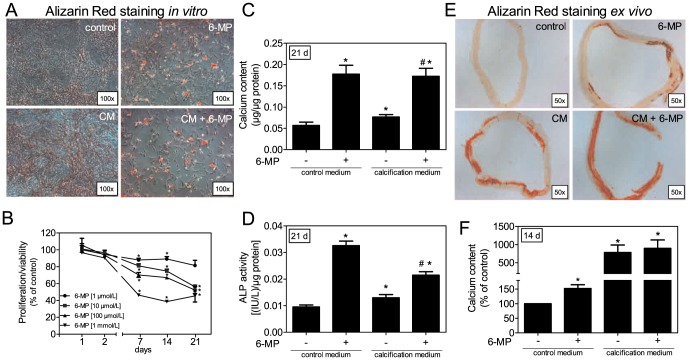
6-MP-induced calcification *in vitro* and *ex vivo*. (A–D) VSMCs were cultured in control medium or CM±6-MP (100 µmol/L) for 21 days. (A) Mineral deposits were visualized via Alizarin red staining. One representative experiment is shown (n = 5). (B) VSMCs were treated with 6-MP (1 µmol/L–1 mmol/L) for up to 21 days and viability/proliferation was measured. (C) Calcium content (n>6) or (D) ALP enzyme activity (n>6) was quantified and normalized to protein content. (E,F) Rat aortic rings were incubated in control medium or CM ± 6-MP (100 µmol/L) for 14 days. (E) One aortic ring treated with each type of stimulation was used for histochemical analysis. Slices were stained with Alizarin Red to visualize calcium deposition. (F) Calcium content was quantified and normalized to the dry weight of aortic rings (n>6). Data represent means±SEM, *p<0.05 vs. control. ^#^p<0.05 vs. CM. ALP: alkaline phosphatase, CM: calcifying medium, 6-MP: 6-mercaptopurine.

Previous studies have reported that medium containing β-glycerophosphate (CM) can induce calcium deposition in VSMCs [15]. This effect can be further increased by co-treatment with dexamethasone (DEX) [9]. We tested other immunosuppressive agents to determine if they could enhance calcium deposition. Cyclosporin A, tacrolimus, and rapamycin did not increase CM-induced calcium deposition after 21 days, whereas co-stimulation with 6-MP and DEX led to a dramatic increase in calcium content (Figure S1). Due to the prominent effect of 6-MP, we further investigated its effect on VSMC mineralization in *in vitro* and *ex vivo* models. 6-MP treatment for 21 days stimulated calcium foci formation, which was visualized via Alizarin Red staining (Figure 1A). Because 6-MP is known to have an anti-proliferative effect on VSMCs [17], and the reduced cell numbers observed with 6-MP treatment (Figure 1A), we measured cell viability/proliferation. Long-term treatment with 6-MP for 7 to 21 days reduced cell number relative to the controls in a dose-dependent manner (Figure 1B). A bar graph with absorbance units for the viability/proliferation assay is included as Figure S2. The calcium content was quantified after decalcification of the cell layer. In this analysis, the calcium content significantly increased upon 6-MP treatment in control media and CM-cultured cells (Figure 1C). In addition, ALP enzyme activity was significantly enhanced by 6-MP compared to cells cultured in either control medium or CM (Figure 1D). Interestingly, the increase in ALP enzyme activity was higher upon 6-MP treatment in control media-stimulated cells than in cells co-stimulated with CM. Therefore, ALP enzyme activity was also measured after 7 days of treatment (Figure S3). Here, 6-MP with or without CM co-treatment induced enzyme activity similarly. To minimize species-specific effects, we also tested the hypothesis on VSMCs from human donors. In human VSMCs, ALP enzyme activity increased upon 6-MP treatment (Figure S4). To test whether these effects could also be verified in an *ex vivo* model, we incubated aortic rings from rats in either control medium or CM in the presence or absence of 6-MP (Figure 1E, F). Histological staining of aortic slices with Alizarin Red revealed mineral deposition in the vessel media (Figure 1E). 6-MP significantly increased the calcium content of the aortic rings (Figure 1F).

### 6-MP promotes expression of osteogenic proteins

**Figure 2:**
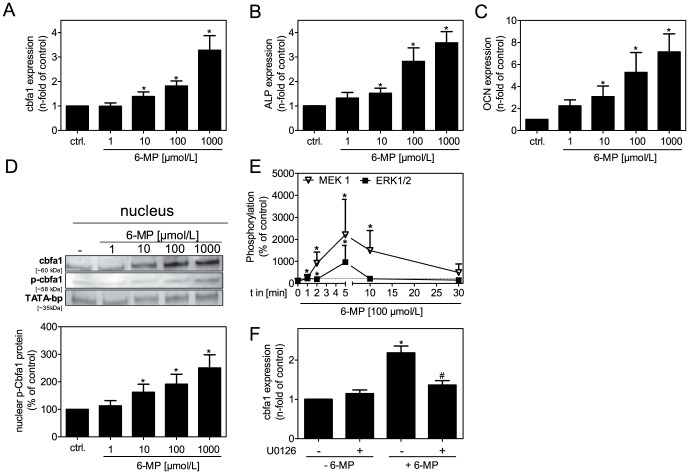
mRNA expression of osteogenic proteins. (A–C) VSMCs were stimulated with 6-MP as indicated and mRNA expression was detected after 48 h. Data represent means±SEM, n≥6,*p<0.05 vs. control. (D) VSMCs were stimulated with 6-MP for 48 h. Nuclear proteins were extracted. Cbfa1, Cbfa1-phospho and TATA-bp were detected via Western blot. Representative images and relative band intensities of 3 independent blots of Cbfa1-phospho are shown. (E) MEK1 and ERK1/2 activation was detected via Bio-Plex (n≥6). Values are given as % of control and are normalized to total kinase. (F) mRNA expression of cbfa1 after 48 h treatment with 6-MP (100 µmol/L) ± U0126 (1 µmol/L) (n>6). Data represent means±SEM, *p<0.05 vs. control. ALP: alkaline phosphatase, cbfa1: core binding factor alpha-1, 6-MP: 6-mercaptopurine, OCN: osteocalcin.

Mineralization of VSMCs is characterized by the expression of osteogenic proteins [9], [15]. We measured the expression of specific genes after stimulation of VSMCs with 6-MP. 6-MP increased the expression of the transcription factor cbfa1 in a dose-dependent manner (Figure 2A). Furthermore, downstream gene products of this transcription factor, such as ALP and osteocalcin (OCN), were also induced by 6-MP (Figure 2B,C). Previous studies have noted that cbfa1 is not only an initial transcription factor in osteoblast differentiation but is also necessary for trans-differentiation of VSMCs into calcifying cells [18]. Therefore, protein expression in VSMCs was also investigated. Cbfa1 activity and DNA binding depend on phosphorylation. Therefore, we measured Cbfa1 in the nuclear protein fraction upon 6-MP treatment of VSMCs. 6-MP increased Cbfa1 and Cbfa1-phospho contents in the nucleus in a dose-dependent manner. TATA-binding protein was used as a loading control (Figure 2D). The bar graph in Figure 2D shows the quantification of band intensity from 3 independent Western blots of Cbfa1-phospho. Previous studies have shown that the mitogen-activated kinases (MAPKs) MEK1 and ERK1/2 can phosphorylate Cbfa1 [19]. Therefore, we investigated the effect of 6-MP on MAPK phosphorylation. Phosphorylation of MEK1 and ERK1/2 was induced in a time-dependent manner upon 6-MP treatment (Figure 2E). Inhibition of the kinases by U0126 diminished cbfa1 expression (Figure 2F).

### Effects of 6-MP metabolites

**Figure 3:**
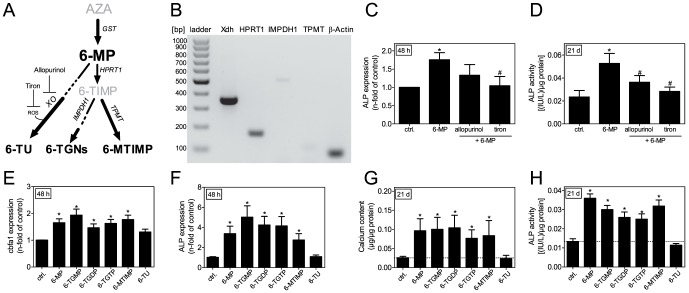
Effects of 6-MP metabolites. (A) Metabolism scheme of the prodrug AZA. (B) Expression of Xdh (351 bp), HPRT1 (168 bp), IMPDH1 (505 bp), TPMT (104 bp), and β-actin (76 bp) in unstimulated VSMCs. (C,D) Effect of 6-MP (100 µmol/L), allopurinol (5 µmol/L) and tiron (100 µmol/L) stimulation on VSMC (C) ALP mRNA expression and (D) ALP enzyme activity. Enzyme activity was normalized to the protein content of the cells. Data represents means±SEM, n≥6, *p<0.05 vs. control ^#^p<0.05 vs. 6-MP. (E,F) VSMCs were stimulated with 6-MP (100 µmol/L), 6-TGNs (each 10 µmol/L), 6-MTIMP (10 µmol/L) or 6-TU (10 µmol/L) for 48 h and (E) cbfa1, and (F) ALP mRNA expression levels were analyzed. Data represents means±SEM, n≥6, *p<0.05 vs. control. (G,H) VSMCs were incubated as indicated (100 µmol/L 6-MP, 6-TGNs [each 10 µmol/L], 6-MTIMP or 6-TU [each 10 µmol/L] for 21 days) and (G) calcium content and (H) ALP enzyme activity were measured. Data represents means±SEM, n≥6,*p<0.05 vs. control. AZA: azathioprine, cbfa1: core binding factor alpha-1, GST: glutathione S-transferase, HPRT: hypoxanthine guanine phosphoribosyltransferase, IMPDH1: inosine monophosphate dehydrogenase 1, 6-MP: 6-mercaptopurine, 6-MTIMP: 6-methylthioinosine monophosphate, 6-TGDP: 6-thioguanosine diphosphate, 6-TGMP: 6-thioguanosine monophosphate, 6-TGNs: 6-thioguanine nucleotides, 6-TGTP: 6-thioguanosine triphosphate, 6-TIMP: 6-thioinosine monophosphate, TPMT: thiopurinemethyltransferase, 6-TU: 6-thiouric acid, Xdh: Xanthine dehydrogenase, XO: xanthine oxidase.

6-MP is a cleavage product of azathioprine (AZA), and this cleavage quickly occurs either non-enzymatically [20] or enzymatically via glutathione S-transferase (GST) [21]. Subsequently, different enzymatic pathways lead to further conversion of 6-MP, including the xanthine oxidase (XO), thiopurinemethyltransferase (TPMT) and hypoxanthine guanine phosphoribosyltransferase (HPRT1) pathways (Figure 3A) [22]. XO metabolizes more than 80% of 6-MP into 6-thio-uric acid (6-TU), and the remaining amount can be further catabolically and anabolically converted into metabolites [23]. The required enzymes for 6-MP metabolism are expressed in rat VSMCs (Figure 2B). Because the majority of 6-MP is metabolized via XO [21], we blocked this pathway and measured the effect on mineralization. As XO is a known source of reactive oxygen species (ROS), we used the inhibitors allopurinol (XO inhibitor) and tiron as scavengers for ROS [24]. To examine the effect of XO inhibition in the mineralization process, we used both inhibitors during co-stimulation with 6-MP. Allopurinol treatment resulted in a reduction of ALP expression and significantly diminished its enzymatic activity, while co-treatment with tiron significantly diminished both ALP expression and enzyme activity (Figure 3C,D).

To determine the effect of 6-MP metabolites on calcification of VSMCs, we examined the three main metabolites of 6-MP: 6-TU, 6-thio-guanine nucleotides (6-TGNs) and 6-methyl-thio-inosine monophosphate (6-MTIMP). All investigated 6-TGNs (6-T-GMP, -GDP, -GTP) and 6-MTIMP, significantly induced cbfa1 and ALP mRNA expression, whereas 6-TU produced no change in expression (Figure 3E,F). This phenomenon could be verified by measuring calcium content and ALP activity after 21 days in culture. With the exception 6-TU, all investigated compounds (6-TGNs and 6-MTIMP) robustly induced calcium deposition (Figure 3G) and ALP enzyme activity (Figure 3H).

### 6-MP promotes the production of ROS

**Figure 4:**
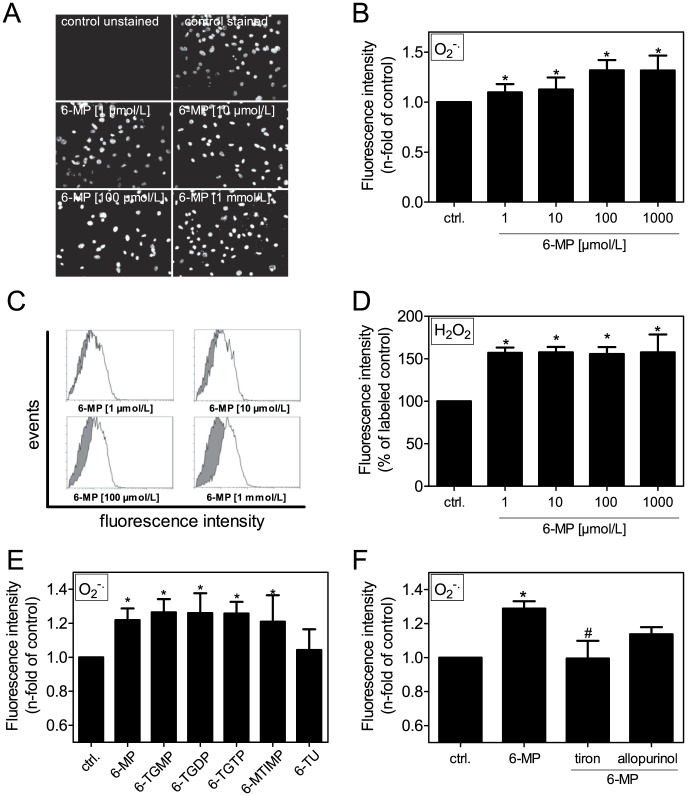
Involvement of ROS. (A,B,E,F) VSMCs were stimulated as indicated for 30 min before labeling cells with DHE. Superoxide production was (A) visualized via fluorescence microscopy (representative images from 3 independent experiments) or (B,E,F) quantified in a fluorescence plate reader (n≥6). (C,D) Hydrogen peroxide production is measured in H_2_DCFDA-labeled cells via flow cytometry. (C) Representative histograms of flow data (grey: control, white: 6-MP). (D) Quantification of fluorescence intensity by % of labeled control (n≥6). (E) Stimulation with 6-MP (100 µmol/L) or its metabolites (each 10 µmol/L). (F) Stimulation with 6-MP (100 µmol/L) alone or costimulation with inhibitors (tiron [1 mmol/L] and allopurinol [1 µmol/L]). DHE: dihydroethidium, 6-MP: 6-mercaptopurine, 6-MTIMP: 6-methylthioinosine monophosphate, 6-TGDP: 6-thioguanosine diphosphate, 6-TGMP: 6-thioguanosine monophosphate, 6-TGN: 6-thioguanine nucleotide, 6-TGTP: 6-thioguanosine triphosphate, 6-TU: 6-thiouric acid.

6-TU is the main metabolite of 6-MP conversion. Inhibition of this pathway by allopurinol and tiron reduced mineralization, but 6-TU itself seemed to have no effect on calcification. Therefore, the metabolic pathway and associated ROS production might be relevant. Previous evidence suggests that the ROS level might contribute to VSMC mineralization [7], [8]. Here, we used several specific probes, in particular superoxide and hydrogen peroxide, to detect ROS levels after treatment of VSMCs with 6-MP and its metabolites. 6-MP increased the production of superoxide in dihydroethidium (DHE)-labeled cells in a dose-dependent manner, as shown by representative fluorescence microscopy images (Figure 4A) and quantification using a 96-well assay (Figure 4B). Superoxide is rapidly converted to hydrogen peroxide. Therefore, we measured its production via flow cytometry (Figure 4C,D). Stimulation of cells with the 6-TGNs and 6-MTIMP also significantly increased superoxide production, whereas 6-TU seemed to have no effect on superoxide production (Figure 4E). Inhibition of XO by allopurinol or tiron treatment diminished the 6-MP-induced production of superoxide (Figure 4F).

## Discussion

In this study, we report that 6-MP, a cleavage product of the immunosuppressive drug AZA, is a potent inducer of vascular calcification in *in vitro* and *ex vivo* models. 6-MP stimulated trans-differentiation of VSMCs into an OCC phenotype, which has been proposed as one hypothesis for vascular mineralization [18]. The generation of ROS by 6-MP could contribute to the mechanism responsible for the trans-differentiation of VSMCs.

These data unmask a potential harmful effect of 6-MP treatment for vascular disease and specifically focus on the progression of the vascular calcification observed in arteriosclerosis. AZA is commonly used, as it is a potent drug that controls many immune diseases [25], [26]. During the last several years, AZA treatment has been replaced in patients who have undergone solid organ transplantation [20], [25]. However, AZAs still routinely used in the treatment of several auto-immune diseases [26]–[28]. Currently, its application as an immunosuppressive drug has increased due to its beneficial effects on lipid profiles and fibrinolytic parameters [12], [29], [30]. However, there are several indications that the cardiovascular risk in AZA-treated patients is increased [27],[28], and vascular alterations could occur. The present study gives additional indications of the potentially harmful effects.

Some clinical studies have revealed relevant roles for immunosuppressive drugs on cardiovascular risk profiles [1], [12], [13], [31]. Reasons for the increased cardiovascular morbidity and mortality are multi-factorial, but there are clear reports that immunosuppressive drugs used to control the aforementioned diseases might influence the occurrence of cardiovascular events [12]. Vascular alterations and the incidence rates of non-fatal and fatal cardiovascular events are some of the major complications in different patient cohorts, such as patients suffering from rheumatoid arthritis (RA) or systemic lupus erythematosus (SLE) [32], dialysis patients or patients who have undergone solid organ transplantation, especially kidney transplant patients (KTX) [1], [2], [33]. Upon AZA treatment, the risk of coronary heart disease increases in patients with SLE [27] and in KTX [31] and RA patients [28].

Generally, it has been thought that the underlying disease might induce a high cardiovascular burden in these patient groups. However, given the present results, it is possible that AZA contributes to the higher cardiovascular risk. One report states that AZA may have atheroprotective properties [34]. In this study, the authors demonstrated that in a hypercholesterolemic apoE transgenic mouse model, local delivery of 6-MP reduced atherosclerosis, mainly due to reduced activation of monocytes [34]. This effect has not yet been demonstrated by systemic application. Studies in dialysis [35], [36] and KTX patients [37]–[39] have shown that vessel calcification due to arteriosclerosis, rather than atherosclerosis, strongly predicts all-cause mortality and cardiovascular disease.

The results of the present study demonstrate that 6-MP induces the expression of typical bone cell markers that induce mineralization in VSMCs. To our knowledge, this report is the first that examines the role of 6-MP with respect to vascular calcification. In the *in vitro* model used, 6-MP produces a stronger effect on calcium deposition in compared with DEX treatment, which has demonstrated a calcification-inducing effect in previous experimental studies [9]. This effect could be verified by measuring the calcium deposition and enzyme activity of ALP in rat and human VSMCs. Similarly, 6-MP treatment results in mineralization of the vessel media in *ex vivo* experiments. The less potent effect of 6-MP in aortic rings compared with its effect *in vitro* might be due to experimental differences between isolated VSMCs and VSMC tissue cells. To verify the observed effects of 6-MP, the expression levels of certain proteins were investigated. The osteogenic transcription factor, cbfa1, and downstream osteogenic proteins, such as OCN, were induced by 6-MP treatment. Cbfa1 is an initial transcription factor in osteogenic differentiation and also appears to play a relevant role in VSMC transformation [18]. We found evidence of induction of the Cbfa1 protein and a phospho variant in VSMCs. Inhibition of ERK1/2 led to decreased cbfa1 mRNA expression. This results supports the known auto-regulatory function of cbfa1 expression [40].

AZA is rapidly converted to 6-MP either non-enzymatically [20] or enzymatically, via GST [21]. Subsequently, 6-MP is enzymatically cleaved into three main metabolites, including 6-TU, 6-MTIMP and 6-TGNs (Figure 3A), which act as pharmacological agents [21]. Here, we investigated these three main metabolites. The investigated concentrations of all metabolites were similar, as expected in the humans after AZA treatment [22]. All 6-TGNs and 6-MTIMP significantly induced osteogenic expression and VSMC mineralization, as detected by calcium deposition and ALP activity. The main metabolite of 6-MP is 6-TU (>80%) [23]. As we have shown here, 6-TU had no effect on vascular calcification, although inhibition of the metabolism of 6-MP to 6-TU via XO reduced calcification. This observation led us to the question, if ROS production, via XO, might be involved [24], [41]. ROS is capable of inducing vascular mineralization [8], [42], [43]. A very similar effect has been previously observed in which activation of XO resulted in the progression of vascular cells to calcifying cells [42]. Furthermore, previous reports show that AZA/6-MP influences oxidative stress [44], [45]. 6-MP is degraded to 6-TGNs and other metabolites, which, with the exception of 6-TU effect vascular calcification and oxidative stress. ROS might contribute to various mechanisms that induce the mineralization phenotype of VSMCs. Further studies investigating the downstream signaling pathways are necessary to describe the effects of the different 6-MP metabolites on vascular calcification.

In conclusion, this study provides evidence that 6-MP intake over an extended period of time in patients who undergo organ transplantation or suffer from autoimmune diseases might have harmful effects that may contribute to the progression of vascular mineralization. Nonetheless, the study has some limitations. We used *in vitro* and *ex vivo* models to show the impact of 6-MP on vascular calcification. 6-MP has anti-proliferative properties on VSMCs and decreases cell number over time relative to the un-stimulated cells. At concentrations of 6-MP between 100 µmol/L to 1 µmol/L, an inhibition of cell proliferation was observed. Most of the experiments in this study were carried out with an expected non-toxic 6-MP concentration (100 µmol/L). At the current stage, the influence of 6-MP-induced cell death or perhaps cell migration in a calcification nidus on the mineralization process *in vitro*, cannot be excluded. Animal studies and clinical prospective and retro-prospective trials are necessary to describe the effects of long-term treatment with AZA/6-MP. In humans, confounding factors, such as disease duration and disease severity, and other therapeutics might have a major influence on the progression of the calcification process. If this possibility is indeed confirmed, 6-MP therapy in any context should be examined to reduce possible harmful long-term cardiovascular effects.

## Supporting Information

Figure S1 **Influence of immunosuppressive drugs on** ***in vitro*** **mineralization.** rVSMCs were cultured in control medium (white bar graph) or CM (black bar graphs) in the presence or absence of DEX (100 nmol/L), CYA, (100 nmol/L), FK506 (10 nmol/L), RPA (1 nmol/L), and 6-MP (100 µmol/L). Calcium deposition was quantified after 21 days and normalized to protein content of the cells. Data represent means±SEM, n≥6, *p<0.05 vs. control, ^#^p<0.05 vs. CM. CM: calcification medium, CYA: cyclosporin A, DEX: dexamethasone, FK506: tacrolimus, RPA: rapamycin, 6-MP: 6-mercaptopurine, VSMCs: vascular smooth muscle cells.
(JPG)

Figure S2 **Cell viability/proliferation.** rVSMCs were cultured in control medium in the presence or absence of 6-MP (1 µmol/L–1 mmol/L) for 1 to 21 days. Data represent means±SEM, *p<0.05 vs. control, 6-MP: 6-mercaptopurine, VSMCs: vascular smooth muscle cells.
(JPG)

Figure S3 **ALP enzyme activity after 7 days of treatment.** rVSMCs were cultured in control medium or calcification medium in the presence or absence of 6-MP (100 µmol/L). ALP enzyme activity, normalized to protein content, was detected after 7 d of treatment. Data represent means±SEM, n = 8, *p<0.05 vs. control, ^#^p<0.05 vs. CM. ALP: alkaline phosphatase, 6-MP: 6-mercaptopurine, VSMCs: vascular smooth muscle cells.
(JPG)

Figure S4 **Mineralization of human VSMCs.** hVSMCs were cultured in control medium or calcification medium in the presence or absence of 6-MP (100 µmol/L). ALP enzyme activity, normalized to protein content of the cells, was detected after 14 d of incubation. Data represent means±SEM, n = 3, *p<0.05 vs. control. ALP: alkaline phosphatase, CM: calcifying medium, 6-MP: 6-mercaptopurine, VSMCs: vascular smooth muscle cells.
(JPG)

Methods S1 (PDF)

Results S1 (PDF)

References S1 (PDF)

Table S1 **Primer sequences.**
(PDF)
