# Supplementary figures and images for: Harmful Effects of the Azathioprine Metabolite 6-Mercaptopurine in Vascular Cells: Induction of Mineralization
Source: PLoS One. 2014 Jul 16;9(7):e101709. doi: 10.1371/journal.pone.0101709 (PMC4100760; doi:10.1371/journal.pone.0101709)

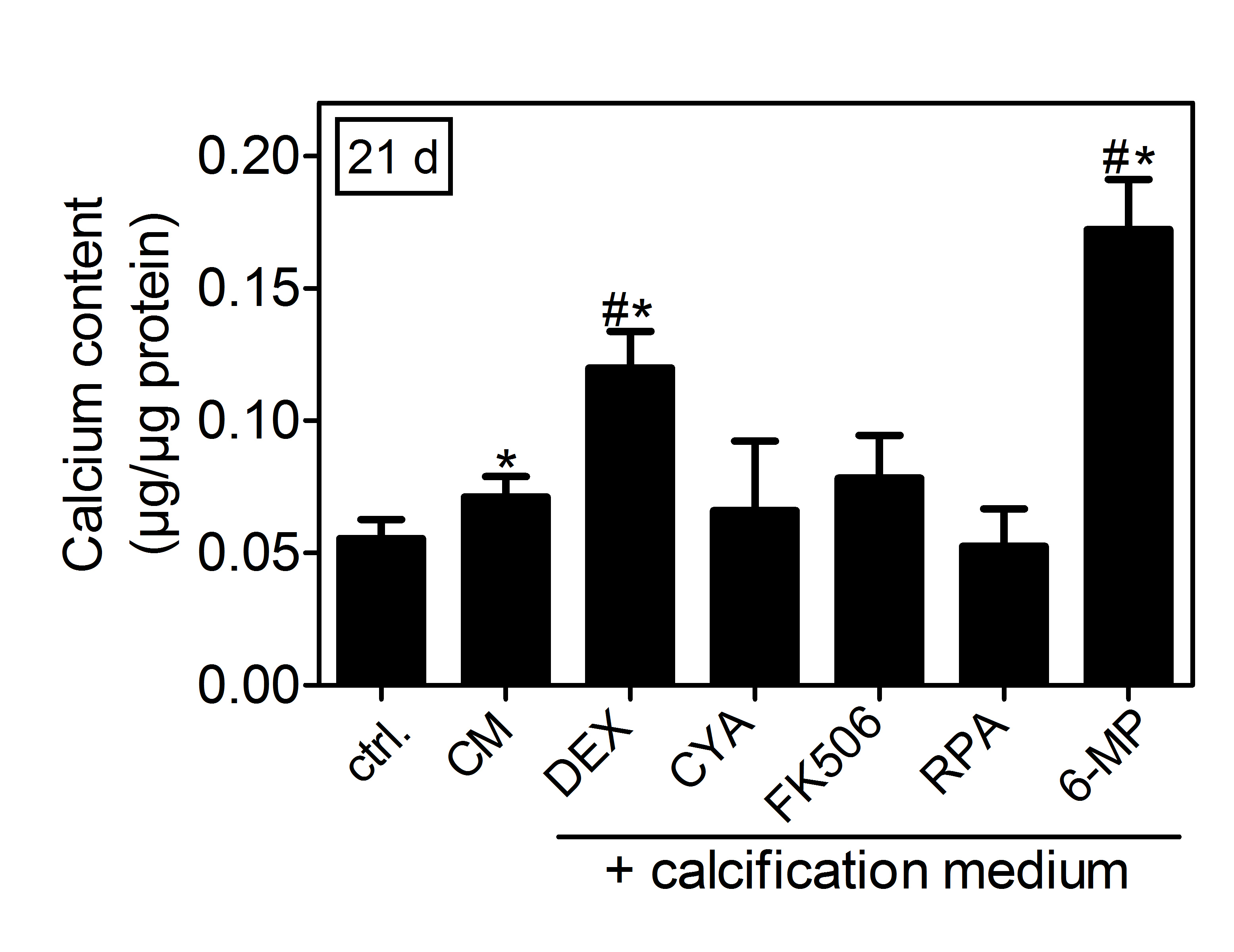

Supplement: Figure S1 — Influence of immunosuppressive drugs on in vitro mineralization. rVSMCs were cultured in control medium (white bar graph) or CM (black bar graphs) in the presence or absence of DEX (100 nmol/L), CYA, (100 nmol/L), FK506 (10 nmol/L), RPA (1 nmol/L), and 6-MP (100 µmol/L). Calcium deposition was quantified after 21 days and normalized to protein content of the cells. Data represent means±SEM, n≥6, *p<0.05 vs. control, #p<0.05 vs. CM. CM: calcification medium, CYA: cyclosporin A, DEX: dexamethasone, FK506: tacrolimus, RPA: rapamycin, 6-MP: 6-mercaptopurine, VSMCs: vascular smooth muscle cells. (JPG) [file pone.0101709.s001.jpg]

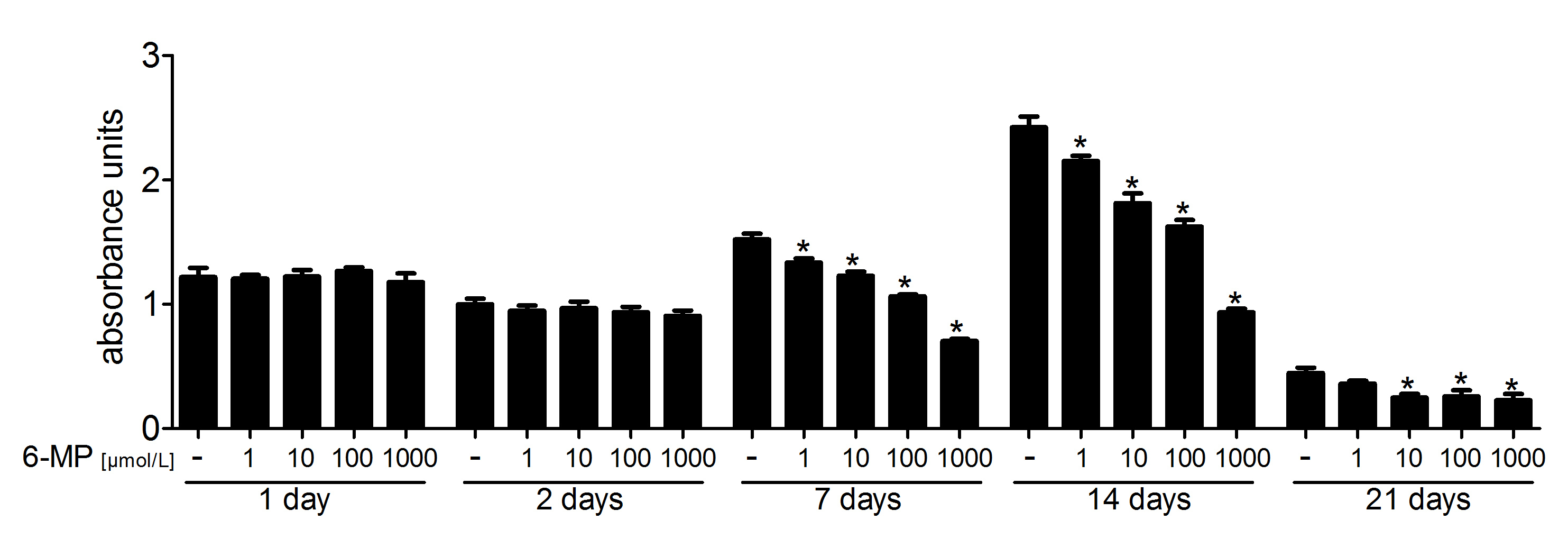

Supplement: Figure S2 — Cell viability/proliferation. rVSMCs were cultured in control medium in the presence or absence of 6-MP (1 µmol/L–1 mmol/L) for 1 to 21 days. Data represent means±SEM, *p<0.05 vs. control, 6-MP: 6-mercaptopurine, VSMCs: vascular smooth muscle cells. (JPG) [file pone.0101709.s002.jpg]

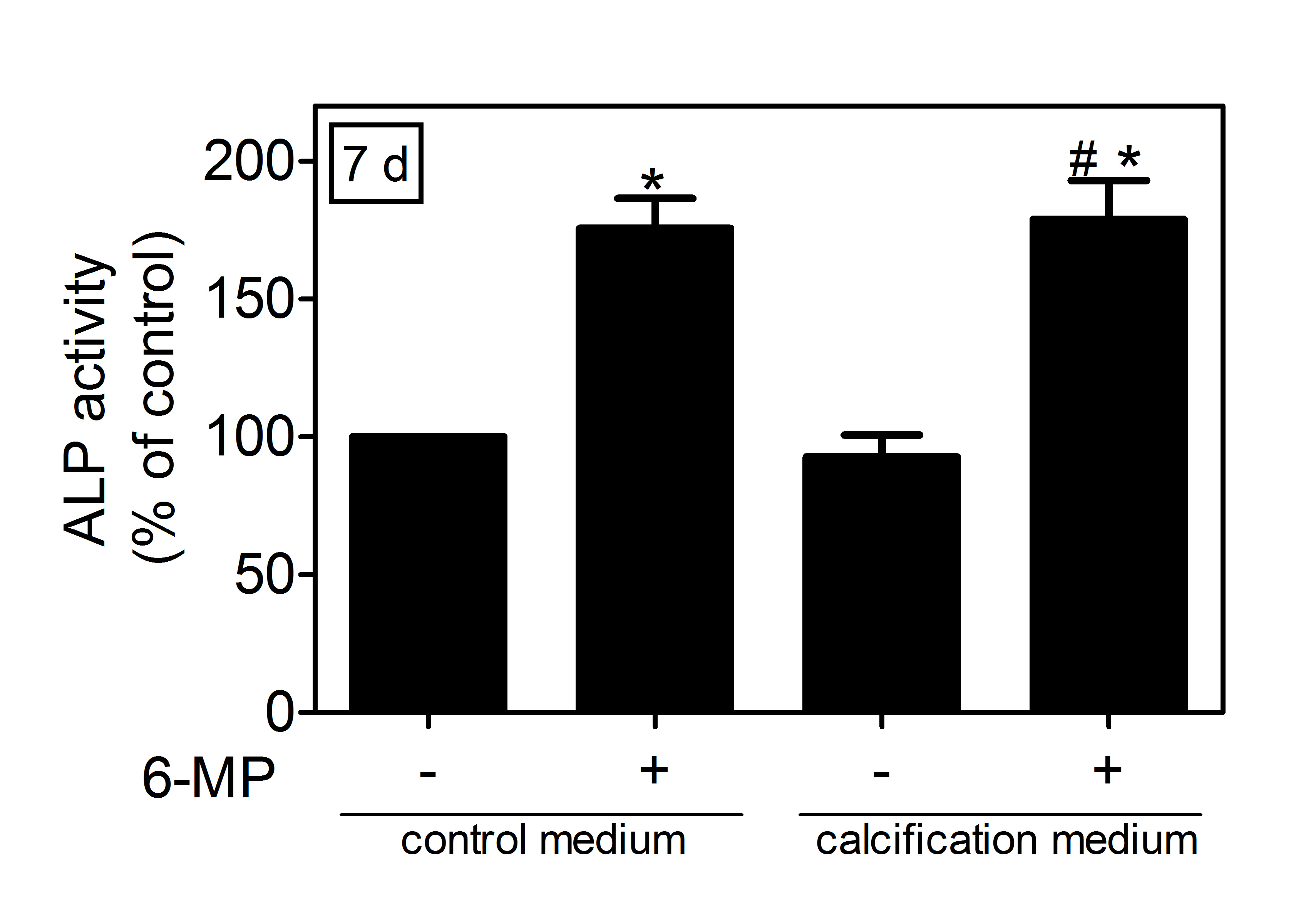

Supplement: Figure S3 — ALP enzyme activity after 7 days of treatment. rVSMCs were cultured in control medium or calcification medium in the presence or absence of 6-MP (100 µmol/L). ALP enzyme activity, normalized to protein content, was detected after 7 d of treatment. Data represent means±SEM, n = 8, *p<0.05 vs. control, #p<0.05 vs. CM. ALP: alkaline phosphatase, 6-MP: 6-mercaptopurine, VSMCs: vascular smooth muscle cells. (JPG) [file pone.0101709.s003.jpg]

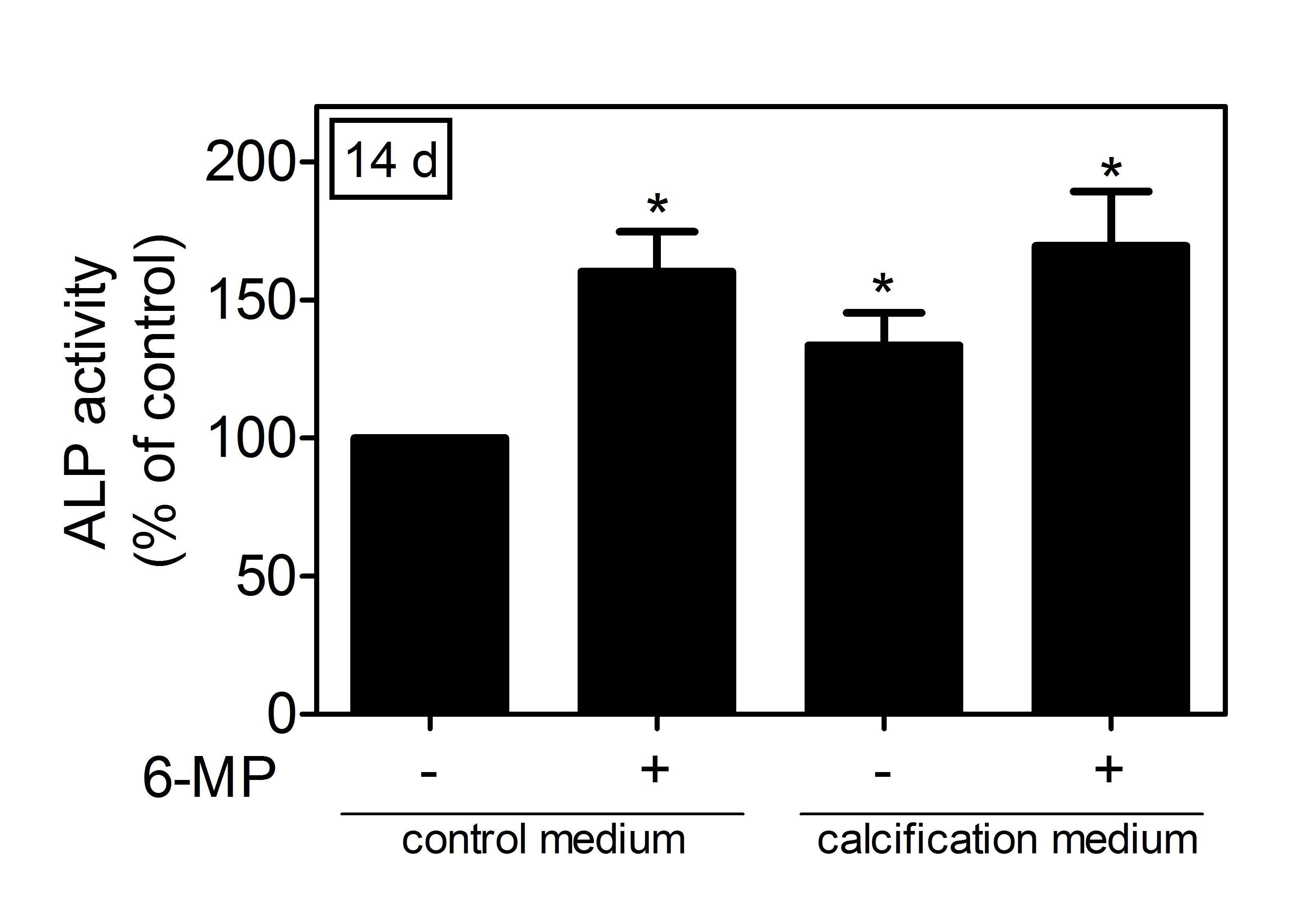

Supplement: Figure S4 — Mineralization of human VSMCs. hVSMCs were cultured in control medium or calcification medium in the presence or absence of 6-MP (100 µmol/L). ALP enzyme activity, normalized to protein content of the cells, was detected after 14 d of incubation. Data represent means±SEM, n = 3, *p<0.05 vs. control. ALP: alkaline phosphatase, CM: calcifying medium, 6-MP: 6-mercaptopurine, VSMCs: vascular smooth muscle cells. (JPG) [file pone.0101709.s004.jpg]
