# Supplementary material for: Harmful Effects of the Azathioprine Metabolite 6-Mercaptopurine in Vascular Cells: Induction of Mineralization
Source: PLoS One. 2014 Jul 16;9(7):e101709. doi: 10.1371/journal.pone.0101709 (PMC4100760; doi:10.1371/journal.pone.0101709)
Supplement: Methods S1 — (PDF) [file pone.0101709.s005.pdf]

Materials and Methods

Materials

All cell culture nutrients were obtained from Biochrom AG. 6-mercaptopurine (6-MP), rapamycin (RPA), dexamethasone (DEX) and all other chemicals were obtained from Sigma-Aldrich unless otherwise indicated. Cyclosporin A (CYA) and tacrolimus (FK506) were bought from LC Laboratories. 6-thio-guanosine monophosphate (6-TGMP), 6-thio-guanosine diphosphate (6-TGDP), 6-thio-guanosine triphosphate (6-TGTP), and 6-methyl-thio-ionosine monophosphate (6-MTIMP) were purchased from Jena BioScience. Thiouric acid (6-TU) was obtained from Santa Cruz.

Cell culture

The isolation of primary rat VSMCs (rVSMCs) by explant outgrowth from thoracic aortas of Wistar rats was done as described previously [1]. Cells were cultured in Dulbeccos Modified Eagle Medium (DMEM) containing 1 g/L glucose supplemented with 10% fetal calf serum (FCS), penicillin (100 U/mL) and streptomycin (0.1 mg/mL). Cells at passages 3 to 8 were used for the present study. Before stimulation, cells were serum-starved for 24 h unless otherwise indicated. In the case of inhibitory experiments, inhibitors were pre-incubated for 30 min before stimulation with drugs. Human VSMCs (hVSMCs) from different donors (n=2, sex: male, age: 19 and 35) were purchased from PromoCell and maintained in low-serum (5% FCS) smooth muscle cell basal medium (SMB) complemented with supplement mix (PromoCell) for a complete growth medium. Cells from passages 5 to 6 were used. All cell types were cultured in a humidified incubator maintained at 37°C with 5% carbon dioxide.

Induction of *in vitro* calcification

For hVSMCs calcification assay, SMB was used as basic media including supplements above containing a lower dose of antibiotics (penicillin 10 U/mL and streptomycin 10 µg/mL).

Alizarin Red staining

Calcium deposition was visualized by Alizarin Red staining as described previously [2-4]. The cells and aortic rings were fixed with 4% buffered formaldehyde. After washing with PBS and distilled deionized water, cells were treated with Alizarin Red solution (2%, pH 4.2) for 20 min, than washed again. Overnight fixed aortic rings were transferred to 70% ethanol and embedded in paraffin via automatic procedure. Tissues were serially cut in 2 µm sections and stained with Alizarin Red solution (0.5%, pH 4.2). Calcification status was imaged using an Axiovert 200M microscope (Zeiss).

Quantification of calcium content

For quantification of mineralization *in vitro*, VSMCs were decalcified in 0.6 mol/L HCl for 24 h. The calcium content was determined by the colorimetrical *o*-cresolphthalein complexone method (Calcium C-test, Wako Chemicals). The remaining cells were washed with PBS and solubilized in cell lysis buffer (0.1 mol/L NaOH/0.1% SDS) and the cell protein content was measured using the bicinchoninic acid (BCA) protein assay kit™ (Pierce). The calcium content was normalized to protein content. The mineralization of aortic rings (*ex vivo*) was quantified using the same method. The calcium content of aortic rings was normalized to dry weight of the tissue.

Measurement of ALP activity

ALP activity of the cells was measured using the ALP Assay Kit from Gentaur utilizing p-nitrophenyl as substrate. To this end, stimulated cells were scraped in 0.2% Triton/phosphate-

buffered saline solution (PBS) containing protease inhibitors (Roche) and protein concentration was determined with BCA protein Assay Kit™ (Pierce).

Polymerase chain reaction (PCR) and agarose gel electrophoresis

Enzyme expression of Xanthine dehydrogenase (Xdh), hypoxanthine guanine phosphoribosyltransferase 1 (HPRT1), inosine monophosphate dehydrogenase (IMPDH1) and thiopurinemethyltransferase (TPMT) was computed using standard PCR and following agarose gel electrophoresis. cDNA was prepared from RNA using 50 U of MultiScribe™ Reverse Transciptase (Applied Biosystems) according to a standard protocol. For specific primers sequences see Table S1. PCR was started at 95°C for 2 min, and subsequent amplification was performed as follows: 95°C for 30s, 60°C for 30 s, 72°C for 1 min and for 35 cycles and 72°C for 5 min in a T100™ thermal cycler (BioRad). Amplification products were loaded on 1.5% ethidium bromide-stained agarose gel. 100 bp ladder (Fermentas) was used. Visualization of PCR products and image acquisition were done in an imaging reader with ChemiCapt software (Vilber Lourmat).

Detection of superoxide

The superoxide formation was assessed by dihydroethidium (DHE, Life Science Technologies). Cells were seeded in clear bottomed 96-well plates (black, Nunc). After 30 min of stimulation, medium was withdrawn and replaced by DHE, diluted in Hank's Balanced Salt Solution (HBSS) to a final concentration of 300 µmol/L kept in the dark until use. The fluorescence increase was measured using the Mithras LB 940 (excitation: 530 nm, emission 620 nm) and analyzed with MikroWin software both from Berthold Technologies. For fluorescence microscopy, cells were seeded in 8-well slides (Ibidi), carefully washed with HBSS after stimulation and fixed with 2% solution of glutaraldehyde for 10 min at room temperature. Afterwards, image acquisition was done using Axiovert 200M microscope with Axio Vision software (Zeiss).

Measurement of hydrogen peroxide

The hydrogen peroxide production (H_2O_2) was determined using the cell-permanent 2',7'-dichlorodihydrofluorescein diacetate (H_2DCFDA) probe. After 24 h of stimulation, cells were washed with PBS and loaded with a 10 mmol/L solution of H_2DCFDA/PBS. Then, H_2DCFDA-loaded cells were incubated at 37°C with 5% CO_2 for 30 min. PBS washed cells were taken in 600 µL of PBS and H_2O_2 production was measured through 2',7'-dichlorofluorescein (DCF) detection via flow cytometry (Beckman Coulter). The data obtained were analyzed with CXP analysis software (Beckman Coulter).

Measurement of gene expression

VSMCs were stimulated for 48 h. After stimulation, cells were washed with ice-cold PBS and scraped and/or lysed into RLT® cell lysis buffer and RNA was isolated according to the RNeasy® Mini kit protocol from Qiagen. From each sample, 500 ng of total RNA was reverse transcribed using the High Capacity cDNA Reverse Transcription Kit from Life Technologies. For determination of mRNA expression, quantitative real-time PCR was performed with detection systems and reagents from Bio-Rad. Primers were synthesized by TibMolBiol. Primer sequences for mRNA expression were used as published earlier [5]. The quantification of mRNA levels was calculated from triplicate analysis of each sample. Obtained mRNA levels for the genes of interest were normalized to ß-actin as housekeeping gene.

Viability/proliferation assay

Measurement of the cell viability/proliferation was done with the CellTiter 96® AQ_{ueous} One Solution Cell Proliferation assay from Promega. VSMCs were plated in 96-well plates and stimulated with 6-MP (1 µmol/L - 1 mmol/L) for 24 - 48 h. After stimulation, viability reagent was added directly to culture wells, incubated for 1 h and then absorbance at 490 nm was measured in a 96-well plate reader (iEMS Reader MF, Thermo Scientific).

Western blot analysis

Protein levels for Cbfa1 and p-Cbfa1 were assessed by Western blotting. After incubation, cells were lysed in NP40 buffer (Life Science Technologies) containing protease inhibitors (Roche). Protein concentrations were determined with the BCA™ protein assay kit (Pierce). Proteins were separated via 12% SDS-PAGE and afterwards transferred to polyvinylidene difluoride (PVDF) membrane using the iblot™-System (Life Science Technologies). After antibody incubation steps, band intensity was detected via chemiluminescence, imaged and quantified as previously described using Bio1D-software (Vilber Lourmat) [5].

For detection of Cbfa1 and p-Cbfa1, VSMCs were starved in serum-free medium for 48 h and were stimulated with 6-MP (1 µmol/L - 1 mmol/L) for 48 h. Cytoplasmatic and nuclear protein extracts were made using NE-PER® Nuclear and Cytoplasmatic Extraction Reagents kit (Pierce) according to the manufacturer's protocol. 10 µg of nuclear extracts were separated and transferred to a membrane. Detection of Cbfa1 was accomplished as previously mentioned [5]. Anti-phospho-Cbfa1 antibody (Abgent, 1:1,000) and anti-TATA-binding protein antibody (1:500) (Thermo Scientific) were used on the same terms. As secondary antibodies anti-mouse IgG (FC) peroxidase antibody (Sigma-Aldrich, 1:5,000) and anti-rabbit IgG peroxidase antibody (KPL, 1:5,000) were used.

Phosphoprotein Detection

After stimulation of VSMCs with 6-MP for the indicated time points, cells were harvested with ice-cold cell lysis buffer (Bio-Rad), centrifuged for 20 min at 4°C and 13,000 rpm, and supernatant was spiked with equal amount of assay buffer (Bio-Rad). Protein concentration of lysates was determined with BCA™ assay kit (Pierce). Determination of phospho as well as total protein was performed using Luminex™ technology with the phospho-protein detection assay in a Bioplex reader (Bio-Rad).

Statistical analysis

Data are presented as means±SEM and compared by Mann-Whitney U test. P values less than 0.05 were considered significant. Calculations and bar graphs were done with GraphPad Prism software (Version 5.0, GraphPad Software).
