# Supplementary material for: Harmful Effects of the Azathioprine Metabolite 6-Mercaptopurine in Vascular Cells: Induction of Mineralization
Source: PLoS One. 2014 Jul 16;9(7):e101709. doi: 10.1371/journal.pone.0101709 (PMC4100760; doi:10.1371/journal.pone.0101709)
Supplement: Results S1 — (PDF) [file pone.0101709.s006.pdf]

Results

Effect of immunosuppressive agents on *in vitro* calcification

As known from literature, ß-glycerophosphate containing medium (CM) induces calcium deposition in VSMCs [6] and this effect could be increased using co-treatment with dexamethasone (DEX) [7]. Beside these, we tested further immunosuppressive agents like cyclosporin A (CYA), tacrolimus (FK506), rapamycin (RPA), and 6-mercaptopurine (6-MP). Out of these, 6-MP has the strongest effect on *in vitro* mineralization, detected via calcium content (Figure S1).

Viability/proliferation assay

The cell viability/proliferation of the cells were measured after treatment with 6-MP for 1 to 21 days. 6-MP treatment reduced the proliferation of the cells in a time and dose-dependent manner (Figure 1B, the data is presented as % of control). The respective absorbance units is given in Figure S2.

ALP enzyme activity after 7 days of treatment

In Figure 1, ALP enzyme activity was detected after 21 days of treatment. To test whether enzyme activity is higher at earlier stages, it is also measured after 7 days. 6-MP and 6-MP in co-stimulation with calcification medium significantly increased ALP enzyme activity (Figure S3).

Effects of 6-MP on calcification in hVSMCs

To exclude species-specific effects of 6-MP treatment, we verified its effect in hVSMCs from two different human donors in three independent experiments. Upon 6-MP (100 µmol/L) treatment for 14 d ALP enzyme activity increased (Figure S4).
