# Supplementary material for: Harmful Effects of the Azathioprine Metabolite 6-Mercaptopurine in Vascular Cells: Induction of Mineralization
Source: PLoS One. 2014 Jul 16;9(7):e101709. doi: 10.1371/journal.pone.0101709 (PMC4100760; doi:10.1371/journal.pone.0101709)
Supplement: References S1 — (PDF) [file pone.0101709.s007.pdf]

References

1. Ross R, Glomset JA (1973) Atherosclerosis and the arterial smooth muscle cell: Proliferation of smooth muscle is a key event in the genesis of the lesions of atherosclerosis. Science 180: 1332-1339.
2. Kanno Y, Into T, Lowenstein CJ, Matsushita K (2008) Nitric oxide regulates vascular calcification by interfering with TGF- signalling. Cardiovasc Res 77: 221-230.
3. Hale LV, Ma YF, Santerre RF (2000) Semi-quantitative fluorescence analysis of calcein binding as a measurement of in vitro mineralization. Calcif Tissue Int 67: 80-84.
4. Perinpanayagam H, Schneider G, Holtman K, Zaharias R, Stanford C (2004) Altered Cbfa1 expression and biomineralization in an osteosarcoma cell line. J Orthop Res 22: 404-410.
5. Schuchardt M, Tolle M, Prufer J, Prufer N, Huang T, et al. (2012) Uridine adenosine tetraphosphate activation of the purinergic receptor P2Y enhances in vitro vascular calcification. Kidney Int 81: 256-265.
6. Shioi A, Nishizawa Y, Jono S, Koyama H, Hosoi M, et al. (1995) Beta-glycerophosphate accelerates calcification in cultured bovine vascular smooth muscle cells. Arterioscler Thromb Vasc Biol 15: 2003-2009.
7. Mori K, Shioi A, Jono S, Nishizawa Y, Morii H (1999) Dexamethasone enhances In vitro vascular calcification by promoting osteoblastic differentiation of vascular smooth muscle cells. Arterioscler Thromb Vasc Biol 19: 2112-2118.
