# Supplementary material for: Harmful Effects of the Azathioprine Metabolite 6-Mercaptopurine in Vascular Cells: Induction of Mineralization
Source: PLoS One. 2014 Jul 16;9(7):e101709. doi: 10.1371/journal.pone.0101709 (PMC4100760; doi:10.1371/journal.pone.0101709)
Supplement: Table S1 — Primer sequences. (PDF) [file pone.0101709.s008.pdf]

Tables

Table S1: Primer sequences.

Gene	Species	Oligonucleotide sequence (5´- 3´)	[bp]
Xdh	rat	fwd. TGG ACA AGT AGA GGG GGC AT<br>rev. ACA CAG GCG TTT CGG ATC TT	351
HRPT1	rat	fwd. CAG TCC CAG CGT CGT GAT TA<br>rev. TGG CCT CCC ATC TCC TTC AT	168
IMPDH1	rat	fwd. CAT GGA GGA ACC GCT CTC AC<br>rev. CTC AAA CTT CTT GAC CTT CCG C	505
TPMT	rat	fwd. TCC CCT CTC TTT GGA GCA GA<br>rev. ATC TGC ATA GCG GTC ACG AT	104
